# Supplementary material for: Description of an activity-based enzyme biosensor for lung cancer detection
Source: Commun Med (Lond). 2024 Mar 5;4:37. doi: 10.1038/s43856-024-00461-7 (PMC10914759; doi:10.1038/s43856-024-00461-7)
Supplement: Supplementary file 1 — Supplementary Information [file 43856_2024_461_MOESM1_ESM.pdf]

Supplementary Information

Description of an activity-based enzyme biosensor for lung cancer detection.

Paul W. Dempsey[1#], Cristina-Mihaela Sandu[1], Ricardo Gonzalezirias[1], Spencer Hantula[1], Obdulia Covarrubias-Zambrano[2], Stefan H. Bossmann[2], Alykhan S. Nagji[2], Nirmal K. Veeramachaneni[3], Nezih O. Ermerak[4], Derya Kocakaya[4], Tunc Lacin[4], Bedrittin Yildizeli[4], Patrick Lilley[5], Sara W.C. Wen[6], Line Nederby[6], Torben F. Hansen[6], Ole Hilberg[6]

1. Hawkeye Bio, Inc., Torrance, CA, USA
2. University of Kansas Medical Center (KUMC), Kansas City, KS, USA
3. St. Louis University, School of Medicine, MO, USA
4. Marmara University, Istanbul, Turkey
5. Liquid Biosciences, Inc., Aliso Viejo, CA, USA
6. Vejle Hospital, University Hospital of Southern Denmark, Vejle, Denmark.

# Corresponding author: pdempsey@hawkeyebio.com
ORcID: 0009-0003-1936-121X

Description of Supplementary Tables

Name: Supplementary Table 1

Description: Enumeration of number of layers in explosion based turbostratic graphene

Name: Supplementary Table 2

Description: Dynamic light scattering (DLS) and zeta potential measurements of the biosensors

Name: Supplementary Table 3

Description: Carbon, Hydrogen, Oxygen and Nitrogen elemental analysis.

Name: Supplementary Table 4

Description: Colloidal stability of biosensors.

Name: Supplementary Table 5

Description: Standard Curve.

Name: Supplementary Table 6

Description: Coefficient of variation at 1:10 sera input.

Name: Supplementary Table 7

Description: Performance by Stage.

Name: Supplementary Table 8

Description: Precision

Name: Supplementary Table 9

Description: Model for the impact of LEAP triage on LDCT screening.

Supplementary Table 1

Sensor	Image	Frame	# layers	Avg.
BM01	BM01 200kV-300KX-Image6	A1	6	
		A2	11	
		A3	9	
	BM01 200kV-300KX-Image5	A1	8	
		A2	6	
		A3	10	8.3
BM02	BM02 200kV-300KX-Image9	A1	5	
		A2	6	5.5
BM03	BM03 200kV-300KX-Image7	A1	5	
		A2	7	
		A3	9	7.0
BM04	BM04 200kV-300KX-Image12	A1	9	
		A2	9	
		A3	6	8.0
BM05	BM05 200kV-300KX-Image5	A1	6	
		A2	9	
		A3	8	7.7
BM06	BM06 200kV-300KX-Image14	A1	6	
		A2	10	
		A3	7	
	BM06 200kV-300KX-Image7	A1	7	
		A2	5	
		A3	14	8.2
BM07	BM07 200kV-300KX-Image15	A1	5	
		A2	7	
		A3	6	
	BM07 200kV-300KX-Image8	A1	7	
		A2	5	
		A3	10	6.7
BM08	BM08 200kV-300KX-Image8	A1	12	
		A2	10	
		A3	9	
	BM08 200kV-300KX-Image6	A1	8	
		A2	12	
		A3	5	9.3
BM09	BM09 200kV-300KX-Image7	A1	5	
		A2	6	
		A3	5	
	BM09 200kV-300KX-Image6	A1	5	
		A2	7	5.6
BM10	BM10 200kV-300KX-Image14	A1	5	
		A2	5	
		A3	7	
	BM10 200kV-300KX-Image7	A1	6	
		A2	7	
		A3	8	6.3

Supplementary Table1 Cont'd

Sensor	Image	Frame	# layers	Avg.
**BM11**	BM11 200kV-300KX-Image15	A1	5	
		A2	7	
		A3	4	
	BM11 200kV-300KX-Image7	A1	6	
		A2	7	5.8
**BM12**	BM12 200kV-300KX-Image14	A1	5	
		A2	5	5.0
**BM13**	BM13 200kV-300KX-Image6	A1	9	
		A2	8	
	BM13 200kV-300KX-Image7	A1	6	7.7
**BM14**	BM14 200kV-300KX-Image10	A1	12	
		A2	5	
		A3	5	7.3
**BM15**	BM15 200kV-300KX-Image17	A1	5	
		A2	6	
		A3	9	
	BM15 200kV-300KX-Image10	A1	10	
		A2	5	7.0
**BM17**	BM17 200kV-300KX-Image12	A1	6	
		A2	6	
	BM17 200kV-300KX-Image13	A1	10	
		A2	10	
		A3	7	7.8
**BM18**	BM18 200kV-300KX-Image17	A1	4	
		A2	11	
		A3	12	
	BM18 200kV-300KX-Image9	A1	7	
		A2	8	8.4
**BM19**	BM19 200kV-300KX-Image8	A1	7	
		A2	11	
	BM19 200kV-300KX-Image15	A1	5	
		A2	5	
		A3	4	6.4
Total Layer Average			7.2	
SD			2.3	
Number of edge examples			79	
Number of images			32	

Supplementary Table 1: Enumeration of number of layers in explosion based turbostratic graphene: Graphene biosensors were examined using a 200kV Hitachi H8100 thermionic field emission transmission electron microscope at an electron acceleration voltage of 200 kV. TEM images were captured using a normative and standardized electron dose on eucentric specimen stage and a constant defocus value from the carbon-coated surfaces. Images were collected at 300,000x magnification. Numbers of layers were counted on images that showed clear edges.

		DLS		Zeta Potential
	Batch Number	Z-Avg	SD	mV
Graphene (G)				
	G-042721	412		32.9
	G-050721	560		36.7
	G-090221	447		33.3
	Average	473	77	34
Carboxy-Graphene (CG)				
	L1CG1.8-1	798		-22.4
	L1CG1.8-2	370		-26.3
	L1CG1.8-3	457		-25.1
	L1CG1.8-4	471		-2.3
	L1CG1.8-5	307		6.2
	L1CG1.8-6	266		-15.9
	Average	445	191	-14
CG-Polyethylimine (CGP)				
	CGP114-M05-018	755		45
	CGP114-M05-025A	316		30
	CGP114-M05-025B	292		34
	CGP114-M05-025A+B	741		45
	CMG-609	226		46
	CGP-M05-044	245		48
	Average	429	249	41
CGP-Biosensor				
	CMG609 (BM17)	208		32
	M05-061A (BM17)	225		30
	M05-061B (BM17)	284		30
	Average	239	40	31

Supplementary Table 2: Dynamic light scattering (DLS) and zeta potential measurements of the biosensors were performed during assembly using the NanoBrook 90Plus PALS. A 0.03 mg/ml solution of each sensor component was prepared using highly pure water (HPLC water, Fisher Cat#: W7-4). Average size (DLS), standard deviation (SD), and zeta potential surface charge are shown.

	Preparation	C (%)	H (%)	O (%)	N (%)	Replicates
**Lot 1**						
	**Graphene**	100.0%				2
	**Carboxygraphene**	97.2%	0.6%	2.1%	<0.5%	2
	**Polyethyleneimine-functionalized Graphene**	93.0%	1.3%	3.2%	2.5%	2
**Lot 2**						
	**Graphene**	100.0%				
	**Carboxygraphene**	98.4%	1.1%	0.5%	<0.5%	1
	**Polyethyleneimine-functionalized Graphene**	85.4%	2.0%	6.2%	6.3%	1

Supplementary Table 3: *Carbon, Hydrogen, Oxygen and Nitrogen elemental analysis.* The determination of C, H, and N content was conducted on a PerkinElmer 2400 Series II CHNS/O Analyzer. For CHN combustion analysis, the samples are combusted in a chamber at 950 °C in pure oxygen. The combustion products are then separated and analyzed in a thermal conducticvity detector. Oxygen Content analysis was conducted on a Thermo Finnigan Flash 2000 CHNS-O Analyzer. Oxygen was analyzed by pyrolyzing the sample in a helium environment in contact with a nickel-plated carbon catalyst at 1060 °C. The resulting nitrogen, hydrogen, and carbon monoxide gases are then separated and the carbon monoxide is analyzed in a thermal conductivity analyzer. Oxygen content was then calculated from the amount of carbon monoxide produced. Results are normalized to 100% w/w and averaged for replicates.

Biosensor	Interval	Change/Interval (%)
**BM07**	9 months	0%
**BM10**	60 min.	-13%
**BM15**	9 months	-3%
**BM17**	60 min.	-13%
**BM17**	9 months	8.7%

Supplementary Table 4: *Colloidal stability of biosensors*. Solutions of BM07, BM10, BM15 and BM17 biosensors were prepared as working solutions at 25 µg/mL in 5 mM MES pH7.4 containing 155 mM NaCl and 10 µM final concentrations each of $MgCl_2$, $CaCl_2$ and $ZnCl_2$. The working solutions were prepared and sonicated in a waterbath for 1 min per ml of solution. Samples were vortexed once immediately before sampling 2 µL three times. The optimal absorbance was established by measuring the absorbance across 250 nm to 650 nm on a Nanodrop. The samples were then allowed to sit for 1 hour without any agitation. After 60 min, 2 µL samples were taken with a pipette tip submerged to 50% depth in the sensor solution. The absorbance spectrum was repeated on the $time_{60}$ sample. The performance is displayed as a percentage change in absorbance efficiency at OD_{265}.
Alternatively, working solutions were further stored at room temperature for 9 months. After 9 months, the solutions were vortexed and 2 µl samples were measured at OD_{265}. The change in absorbance from $time_0$ is presented as percent change. The analysis shows the stability of sensor reflected in the low change in absorbance over time. This observation is not dependent on the biosensor measured.

Standard Curve										
Standard	Conc. (ng/ml)	Rep 1	Rep 2	Rep 3	Avg. RFU	S.D.	% CV	Corrected RFU	Backfit Conc.	Recovery %
A	800	67.7	66.1	64.3	66.0	1.7	2.6	66.0	800.1	100%
B	400	35.3	34.5	34.1	34.6	0.6	1.8	34.6	399.7	100%
C	200	17.9	18.1	17.5	17.8	0.3	1.7	17.8	199.8	100%
D	100	9.34	9.01	9.03	9.13	0.2	2.0	9.10	100.1	100%
E	50	5.08	4.58	4.67	4.78	0.3	5.6	4.75	51.25	102%
F	25	2.54	2.29	2.50	2.44	0.1	5.5	2.41	25.33	101%
Blank	0	0.026	0.048	0.017	0.031	0.0	51.4	0.0	-1.3	
							R-squared Avg.	0.99997		
							R-squared 1 S.D.	2.7E-05		

Supplementary Table 5: ***Standard Curve.*** A serial dilution of TCPP-peptide conjugate was prepared in assay buffer. Triplicate wells were run with each assay plate and a polynomial standard curve was used to calculate the concentration of TCPP-peptide product in individual biosensor reactions. Across 85 plates, the average R^2 for all standard curves was 0.9999. One example curve is presented showing average relative fluorescence units (RFU) at each dose +/- s.d.

Biosensor	CV%
BM01	4.5%
BM02	6.5%
BM03	8.9%
BM04	8.4%
BM05	8.2%
BM06	3.3%
BM07	5.8%
BM08	4.1%
BM09	3.3%
BM10	6.2%
BM11	7.1%
BM12	7.0%
BM13	7.2%
BM14	5.5%
BM15	3.8%
BM16	5.5%
BM17	4.5%
BM18	5.2%
BM19	8.2%
Average	6.0%

Supplementary Table 6: ***Coefficient of variation at 1:10 sera input***. 14 sera were assayed with triplicate measurements and the variance between the triplicates were evaluated for each biosensor to determine the intra-plate variance. Data presented are for the 50 min measurement.

Supplementary Table 7: Performance by Stage

**All Samples**			Spec Max			Spec Bias			Balanced			Sens Bias			Sens Max		
Stage	*TNM*	Count	No.	Acc (%)	95% CI	No.	Acc (%)	95% CI	No.	Acc (%)	95% CI	No.	Acc (%)	95% CI	No.	Acc (%)	95% CI
IA		75	42	56%	45-66%	44	59%	48-69%	63	84%	74-90%	64	85%	75-91%	72	96%	88-98%
	IA1	*5*	*1*	*20%*		*1*	*20%*		*2*	*40%*		*3*	*60%*		*4*	*80%*	
	IA2	*41*	*25*	*61%*		*26*	*63%*		*34*	*83%*		*34*	*83%*		*39*	*95%*	
	IA3	*29*	*16*	*55%*		*17*	*59%*		*27*	*93%*		*27*	*93%*		*29*	*100%*	
IB		15	8	53%		9	60%		12	80%		12	80%		13	87%	
IIA		7	6	86%	42-74%	6	86%	42-74%	6	86%	65-91%	6	86%	68-93%	7	100%	83-99%
IIB		34	18	53%		18	53%		27	79%		28	82%		32	94%	
IIIA		39	28	72%	52-79%	28	72%	52-79%	32	82%	69-92%	32	82%	69-92%	38	97%	87-100%
IIIB		12	6	50%		6	50%		10	83%		10	83%		11	92%	
IVA		7	6	86%	49-83%	6	86%	52-86%	7	100%	79-99%	7	100%	79-99%	7	100%	89-100%
IVB		24	15	63%		16	67%		22	92%		22	92%		24	100%	
Unknown		7	6	86%	42-100%	6	86%	42-100%	6	86%	42-100%	6	86%	42-100%	7	100%	59-100%
Total		***220***															

**Test Set**			Spec Max			Spec Bias			Balanced			Sens Bias			Sens Max		
Stage	*TNM*	Count	No.	Acc (%)	95% CI	No.	Acc (%)	95% CI	No.	Acc (%)	95% CI	No.	Acc (%)	95% CI	No.	Acc (%)	95% CI
IA		22	12	55%	36-74%	12	55%	36-74%	19	86%	73-98%	20	91%	77-99%	22	100%	88-100%
	1A1	*1*	*0*	*0%*		*0*	*0%*		*0*	*0%*		*1*	*100%*		*1*	*100%*	
	1A2	*9*	*7*	*78%*		*7*	*78%*		*8*	*89%*		*8*	*89%*		*9*	*100%*	
	1A3	*12*	*5*	*42%*		*5*	*42%*		*11*	*92%*		*11*	*92%*		*12*	*100%*	
IB		7	4	57%		4	57%		7	100%		7	100%		7	100%	
IIA		1	1	100%	28-85%	1	100%	28-85%	1	100%	43-95%	1	100%	43-95%	1	100%	62-100%
IIB		11	6	55%		6	55%		8	73%		8	73%		10	91%	
IIIA		10	7	70%	36-83%	7	70%	36-83%	10	100%	73-100%	10	100%	73-100%	10	100%	73-100%
IIIB		8	4	50%		4	50%		7	88%		7	88%		7	88%	
IVA		1	1	100%	36-100%	1	100%	36-100%	1	100%	54-100%	1	100%	54-100%	1	100%	54-100%
IVB		5	4	80%		4	80%		5	100%		5	100%		5	100%	
Unknown		3	3	100%	29-100%	3	100%	29-100%	3	100%	29-100%	3	100%	29-100%	3	100%	29-100%
Total		***68***															

		Precision Performance				
		**Spec Max**	**Spec Bias**	**Balanced**	**Sens Bias**	**Sens Max**
**Entire Dataset:**	*n=750*	93%	97%	91%	89%	89%
**Validation Set:**	*n=250*	94%	96%	98%	98%	90%

Supplementary Table 8: *Precision*. Intra assay precision was assessed by evaluating the frequency that an assay on a single sample repeated on three different days gave the same result. The entire dataset included 150 precision samples that were assessed on three independent assays. The Test set precision was evaluated on 50 Test Set out of sample intra-assay repeats.

Model population of USPSTF Smokers (n=100,000)

0.91% Prevalence	Lung Cancer	Healthy
Positive	True Positive<br>910	
Negative		True Negative<br>99,090

Performance of LEAP test on USPSTF population (n=75,000)

75% LEAP compliant	Lung Cancer (n=682)	Healthy (n=74,318)	
Positive	True Positive<br>612	False Positive<br>13,080	PPV<br>4.5%<br>*4.1-4.7%*
Negative	False Negative<br>70	True Negative<br>61,328	NPV<br>99.9%<br>*99.9-100%*
LEAP Only	Sensitivity<br>89.7%<br>*87.4-92.1%*	Specificity<br>82.4%<br>*81.7-82.3%*	Accuracy<br>82.5%<br>*81.8-82.4%*

LDCT screen of LEAP "Positive" triaged population (n=13,692)

	Lung Cancer (n=612)	Healthy (n=13,080)	
Positive	True Positive<br>520	False Positive<br>1,674	PPV<br>23.7%<br>*21.9-25.5%*
Negative	False Negative<br>90	True Negative<br>11,406	NPV<br>99.2%<br>*99.0-99.4%*
Combined LEAP + LDCT	Sensitivity<br>85.2%<br>*81.9-87.7%*	Specificity<br>87.2%<br>*86.6-87.8%*	Accuracy<br>87.1%<br>*86.5-87.7%*

Screening population with LDCT only (n=3,900)

(3.9% compliant population)	Lung Cancer (n=35)	Healthy (n=3,865)	
Positive	True Positive<br>30	False Positive<br>495	PPV<br>5.7%<br>*3.9-8.1%*
Negative	False Negative<br>5	True Negative<br>3,370	NPV<br>99.9%<br>*99.7-99.9%*
LDCT only	Sensitivity<br>85.7%<br>*69.7-95.2%*	Specificity<br>87.2%<br>*86.3-88.2%*	Accuracy<br>87.2%<br>*86.1-88.2%*

Supplementary Table 9: ***Model for the impact of LEAP triage on LDCT screening.*** A model population of 100,000 USPSTF lung cancer screening population with a baseline penetrance of 0.91% lung cancer. Baseline population (Top left box) exposed to the LEAP test as a triage (Top right box) would enrich cancer subjects assuming NLST based LUNG-RADS performance (Bottom left box). The screening population exposed only to LDCT would result in significantly fewer discovered lung cancers (Bottom right box).

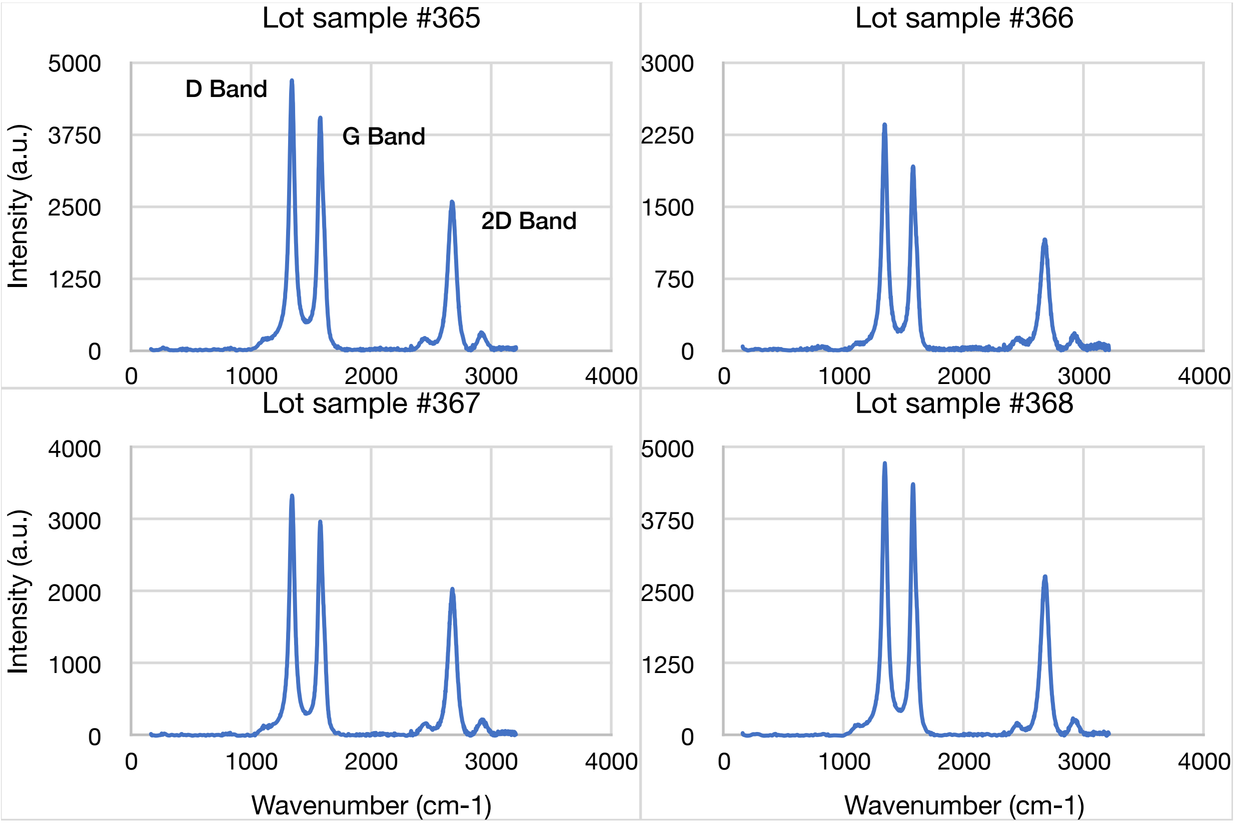

Supplementary Figure 1: *Raman analysis of a sample of graphene-polymer based nanoparticles*.

Raman spectrum of 4 separate lots of detonated graphene powder was measured using the Reinshaw inVia Reflex Raman and a 532nm excitation. Raw dry graphene powders were measured unprocessed and at ambient air and pressure.The Raman spectra of detonated graphene collected with 532 nm excitation are measured. The spectra contained the G band at 1575 cm^{-1}, a strong D band at 1340 cm^{-1} and a symmetrical 2D band at 2680 cm^{-1}, but weaker than the G band. The relative position and sharpness of the G band across the various lots represents consistent multilayer thickness graphene. The presence of a strong D band indicates reduced in-plane sp^2 domains due to the ring breathing mode from sp^2 carbon rings adjacent to graphene edges or defects; the D band is expected to be significant in our detonated graphene powder material. Since 2D band was symmetrical, the sample was not graphite but graphene, which was multilayer graphene since the 2D band was weaker than the G band. The 2D/G intensity ratio also confirms consistent multilayer graphene present in our material. The presence of a sharp and symmetrical 2D band is the result of a two phonon lattice vibrational process always present in graphene and not graphite. The Raman spectra of the sample were typical of multilayer graphene.

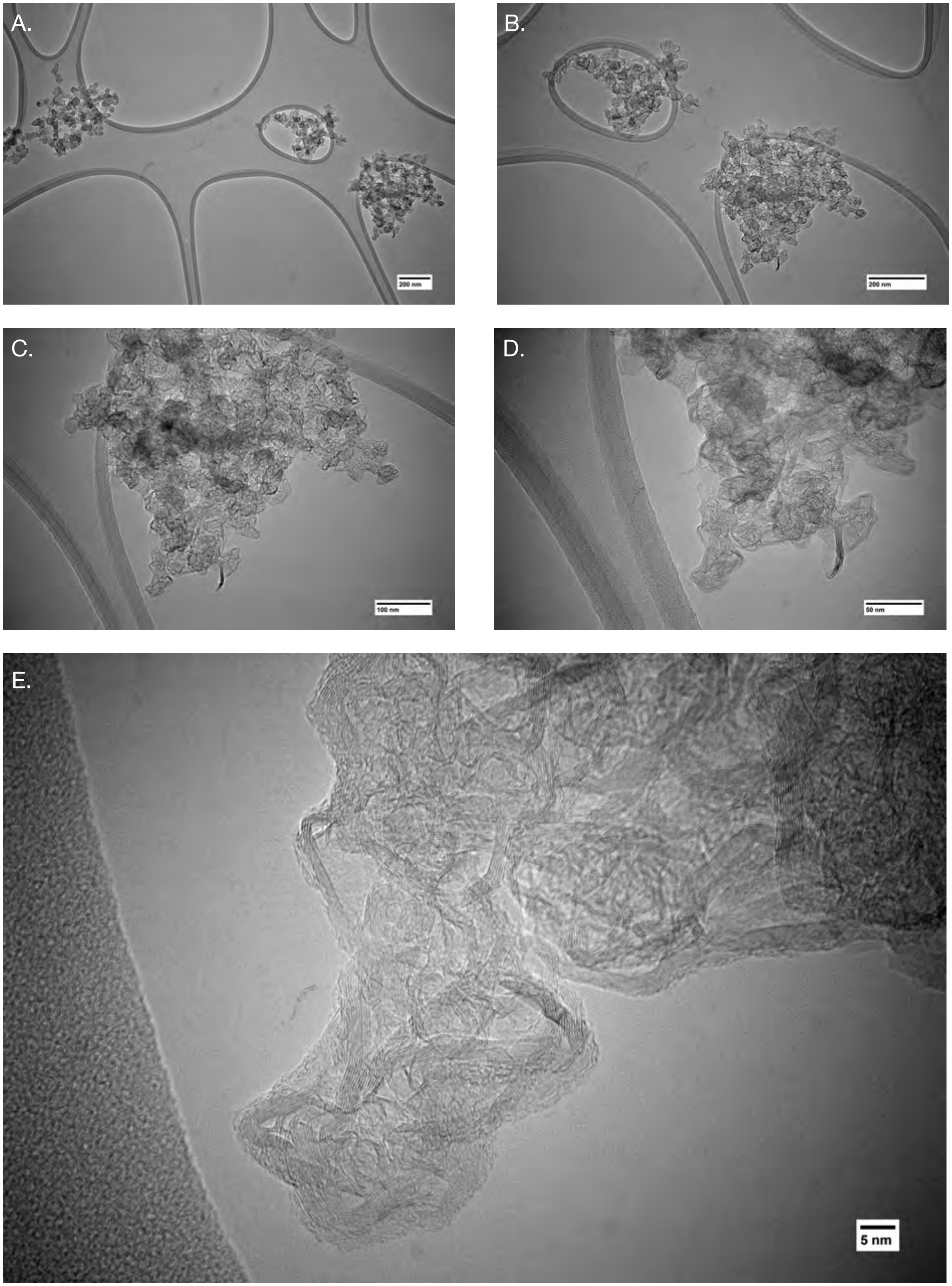

Supplementary Figure 2: ***Representative scanning electron micrographs of graphene biosensor.*** Biosensors were assembled with peptide target for BM10. Scanning electron

micrographs were collected at (A.)15,000x, bar: 200nm, (B) 25,000x, bar: 200nm,(C) 50,000x, bar: 100nm, (D) 100,000x, bar: 50nm, and (E) 300,000x magnification, bar: 5nm. Overall size can be seen and the layered structure of the graphene sheets are revealed. 5 mg of each biosensor was mixed with 1mL of 100% ethanol and sonicated for 5 minutes. 5 μL of diluted biosensor solution was placed on a 300-mesh carbon-coated copper grids and incubated for 1 minute. After incubation, a piece of filter paper was used to wick away the remaining solution and grid was placed onto a clean piece of filter paper to let air dry. The grids with nanobiosensors were examined using a 200kV Hitachi H8100 thermionic field emission transmission electron microscope at an electron acceleration voltage of 200 kV. TEM images were captured using a normative and standardized electron dose on eucentric specimen stage and a constant defocus value from the carbon-coated surfaces. Images were randomly acquired at 10 different locations within the grid.

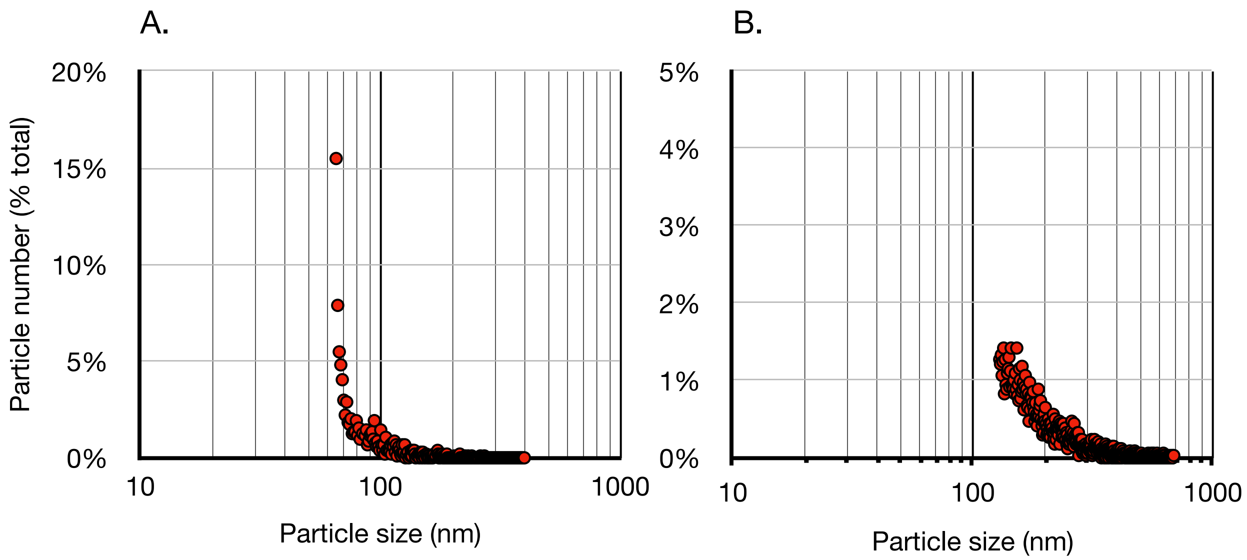

Supplementary Figure 3: ***Microfluidic resistive pulse sensing measuring concentration and size distribution of biosensor particles.*** Sample was prepared by diluting BM17 in filter sterilized phosphate buffered saline and 1% Tween 20 to a final concentration of 150 µg/mL for C-400 cartridge and 1.5 mg/mL for the C-900 cartridge. 3.0 µL of each sample was loaded into a buffer purged cartridge to analyze the particles. Graphs represent measurements using a (A.) C400 cartridge (65-400nm particle diameter particles) and (B.) a C900 cartridge (130-900nm particle diameter). The majority of NBS particles measure between 80-300nm in diameter. Good agreement between cartridge data was observed. The majority of NBS particles measure between 80-300nm in diameter in agreement with DLS observations. The weighted average sizes for the two cartridges was 87nm and 212nm for the C400 and C900 respectively.

Microfluidic Resistive Pulse Sensing measures concentration and size distribution of biosensor particles by electronic, not optical, measurements using the Spectradyne nCS1 C400 cartridge (65-400nm particle diameter) and C900 cartridge (130-900nm particle diameter). Particles are passed through a nano-constriction in the microfluidic device and measured by a change in voltage in the aperture proportional to the particle volume. The Spectradyne nCS1 therefore uses electrical detection to analyze particle concentration of non-sphereical particles between 50nm-900nm in diameter, resulting in more robust particle analysis relative to DLS which relies on spherical approximations.

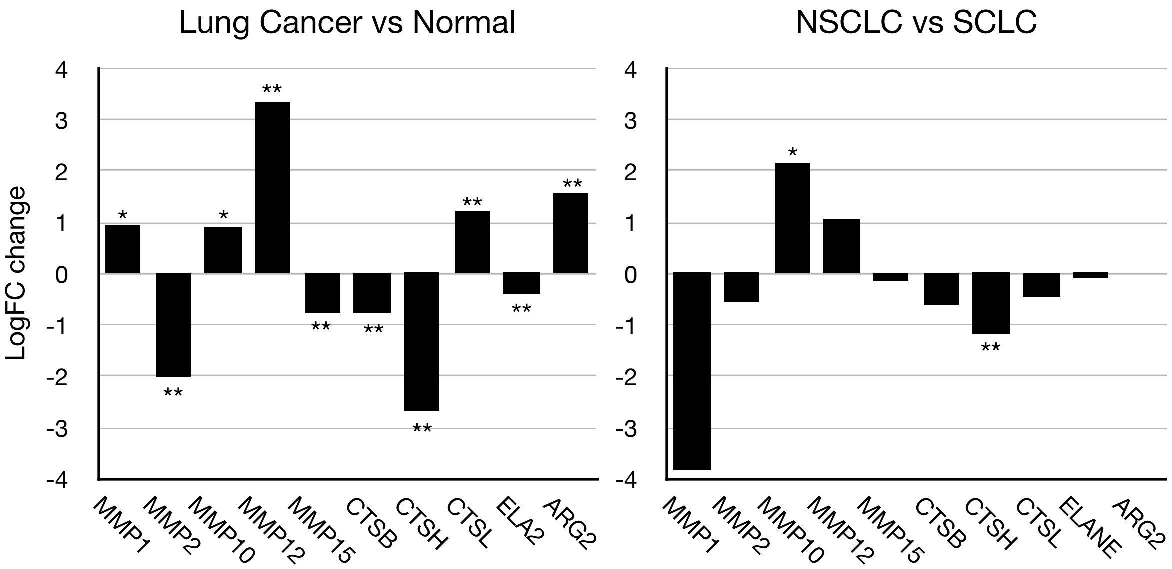

Supplementary Figure 4: ***GEO data sets comparing primary tumor and healthy human tissue***. A search of the GEO Profiles database revealed differences between lung cancer and non lung cancer (GEO accession GDE40275; Kastner *et al*., 2012 [1]. GSE30118; Ihsan *et al.,* 2011 [2]. GSE6044; Rohrbeck *et al.,* 2008 [3]) accessed in October 2020. Graphs showing differentially expressed matrix metalloproteinase and cathepsin enzyme targets including Arginase, as post translational modifiers that distinguish lung cancer and non-lung cancer samples by expression. Also, the same panel might have efficacy distinguishing NSCLS and SCLC based on a similar list. (* $p \leq 0.001$, ** $p \leq 0.0001$) The peptide designed for CTSL was insoluble in several designs and so was not included in the final panel. This list of targets was expanded to include peptides included in previous studies with utility in breast, pancreatic and lung cancers described in Table 1.

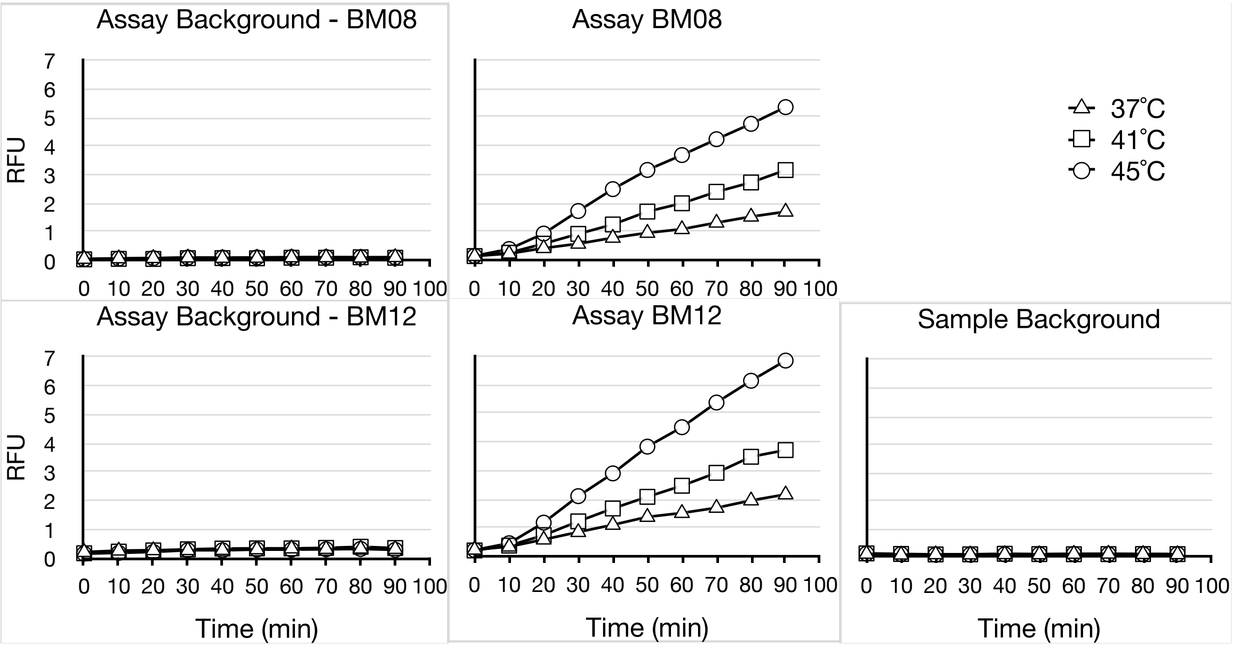

Supplementary Figure 5: ***Increasing temperature of assay incubation increases signal of biosensors.*** 125 µL BM08 and BM12 biosensors were stimulated with 5 µl buffer only (Assay Background - Left) or 5 µl pooled normal human serum (Assay - Middle) and the assay was incubated at 37˚C (triangles), 41˚C (squares) or 45˚C (circles) in the VarioSkan Lux. Fluorescence was measured at 10 minute intervals for the duration of the incubation. Sample Background measurements showed the results from wells that were incubated with 5 µL of serum and 125 µL of buffer but no sensor added (Sample Background - right).

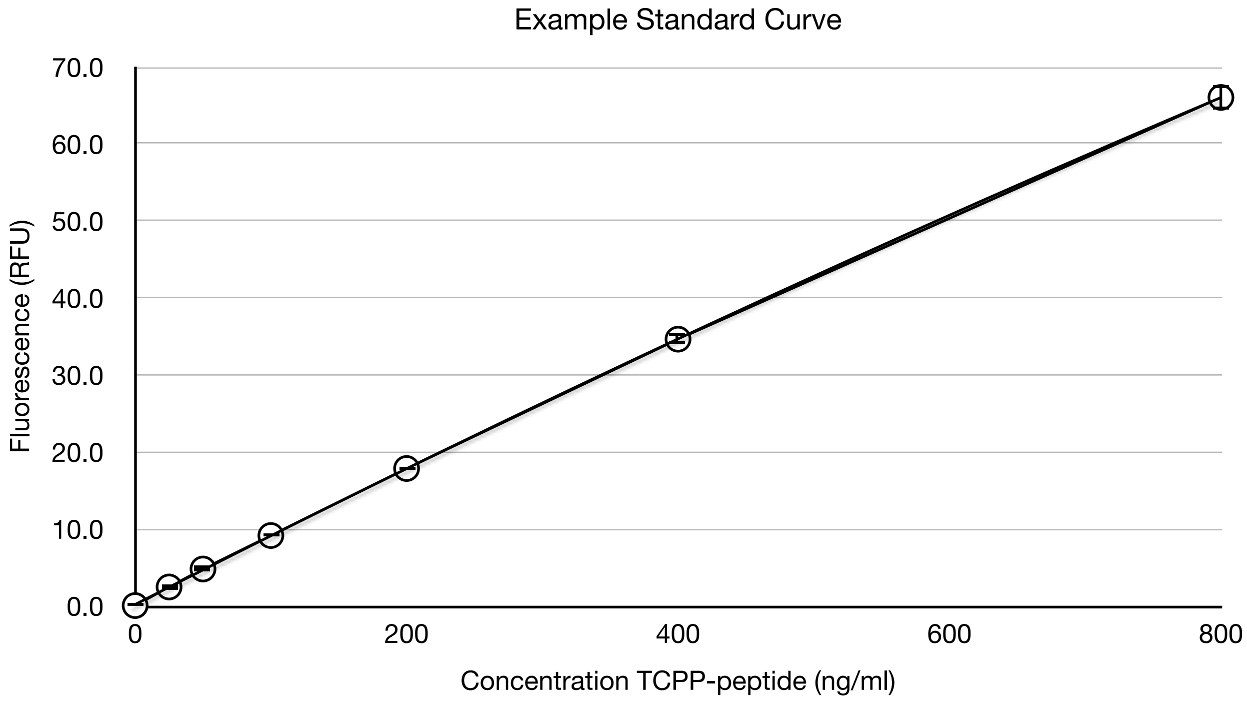

Supplementary Figure 6: ***Standard Curve Performance.*** A serial dilution of TCPP-peptide conjugate was prepared in assay buffer. Triplicate wells were run with each assay plate and a polynomial standard curve was used to calculate the concentration of TCPP-peptide product in individual biosensor reactions. One example curve is presented showing average relative fluorescence units (RFU) at each dose +/- s.d.

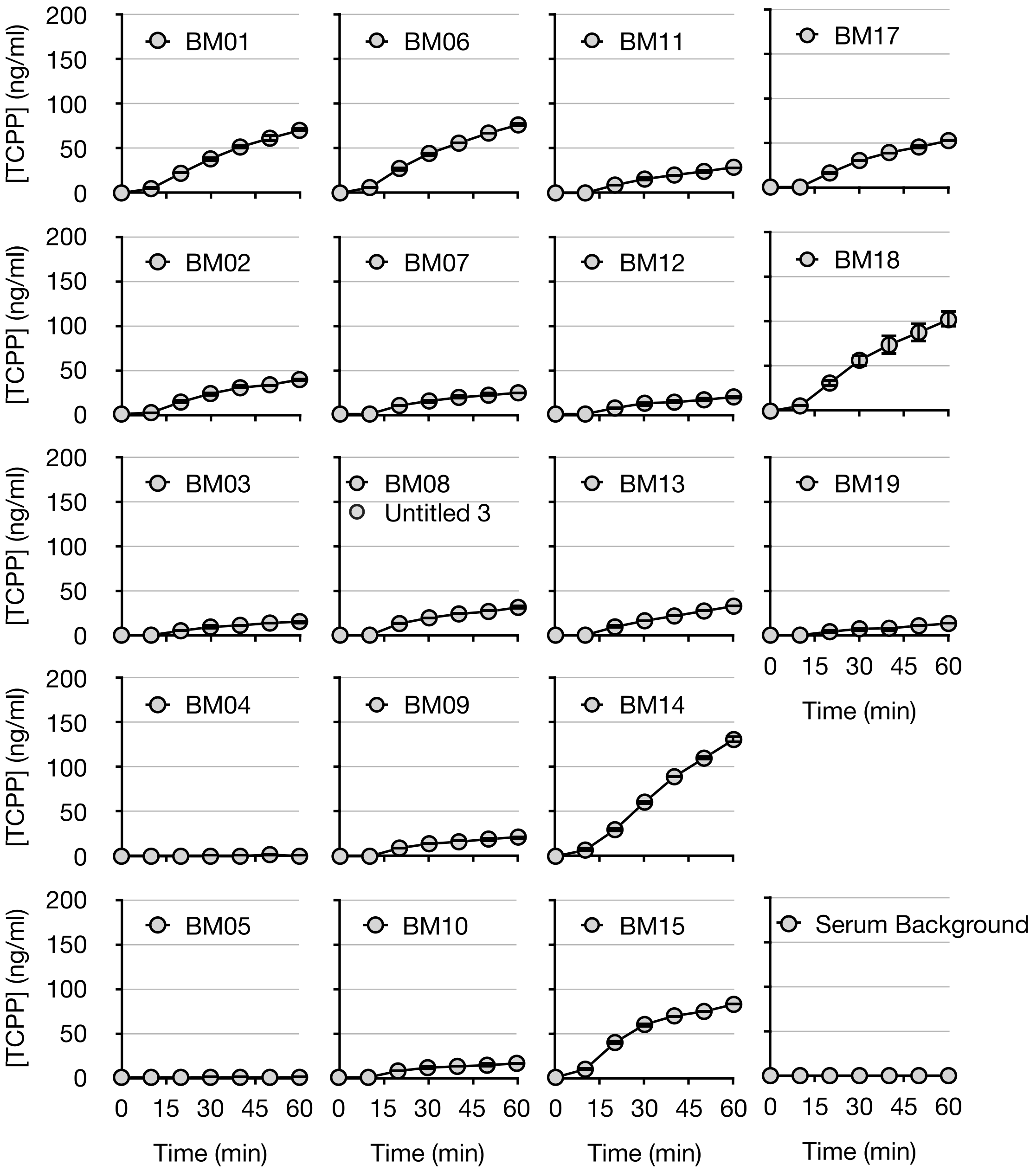

Supplementary Figure 7: ***Biosensor activity profile from normal human serum.*** A panel of biosensors were evaluated by incubating 8 µl serum in an 80 µl reaction containing 25 µg/ml of each biosensor. The reaction was incubated for 60 min at 45˚C and read on a VarioSkan Lux fluorescent plate reader exciting at 422 nm and reading emissions at 650 nm. Graphs show average TCPP concentration at 10 min intervals for all 18 sensors calculated from average of duplicate wells (+/- s.d.).

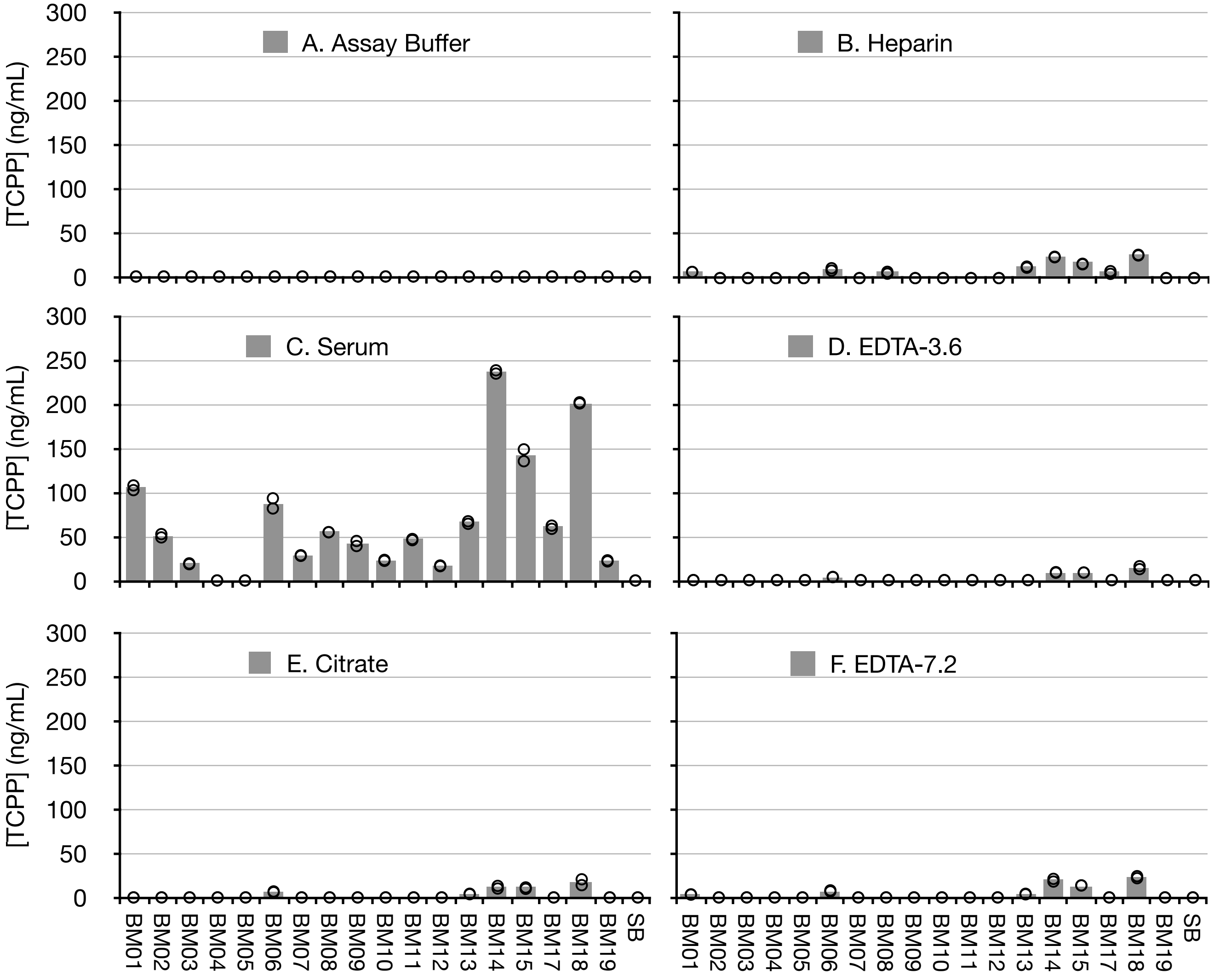

Supplementary Figure 8: ***Biosensor performance with different sample types:*** Blood was collected from a single healthy donor using various blood collection tubes (BCT). A 1:10 dilution of plasma/serum from each BCT was assayed at 45°C for 60 minutes. The concentration of TCPP released is shown for the 60 minute time point only. SB indicates serum only background noise. Sensors were incubated with the following sample types: (A) assay buffer only, (B) Lithium Heparin plasma, (C) serum separator tube, (D) K2 EDTA (3.6 mg), (E) Sodium Citrate, (F) K2 EDTA (7.2 mg). All blood samples were incubated for 30 minutes at room temperature before being spun for 10 minutes at 1300 x g. The serum/plasma fraction was aliquoted and frozen before assay. Graphs display average TCPP released after 60 minutes (bar) and the individual of duplicate readings (open symbol).

Supplementary References

1. Kastner, S., et al., *Expression of G protein-coupled receptor 19 in human lung cancer cells is triggered by entry into S-phase and supports G(2)-M cell-cycle progression.* Mol Cancer Res, 2012. **10**(10): p. 1343-58.
2. Ihsan, R., et al., *Multiple analytical approaches reveal distinct gene-environment interactions in smokers and non smokers in lung cancer.* PLoS One, 2011. **6**(12): p. e29431.
3. Rohrbeck, A., et al., *Gene expression profiling for molecular distinction and characterization of laser captured primary lung cancers.* J Transl Med, 2008. **6**: p. 69.
