# Supplementary material for: Description of an activity-based enzyme biosensor for lung cancer detection
Source: Commun Med (Lond). 2024 Mar 5;4:37. doi: 10.1038/s43856-024-00461-7 (PMC10914759; doi:10.1038/s43856-024-00461-7)
Supplement: Supplementary file 2 — Description of Additional Supplementary Files [file 43856_2024_461_MOESM2_ESM.docx]

**Description of Additional Supplementary Files**

**File Name:** Supplementary Data 1

**Description:** Raw data for primary figures
