# Supplementary material for: Description of an activity-based enzyme biosensor for lung cancer detection
Source: Commun Med (Lond). 2024 Mar 5;4:37. doi: 10.1038/s43856-024-00461-7 (PMC10914759; doi:10.1038/s43856-024-00461-7)
Supplement: Supplementary file 4 — Reporting Summary [file 43856_2024_461_MOESM4_ESM.pdf]

nature portfolio

Corresponding author(s): Paul W. Dempsey

Last updated by author(s): Feb 14, 2024

Reporting Summary

Nature Portfolio wishes to improve the reproducibility of the work that we publish. This form provides structure for consistency and transparency in reporting. For further information on Nature Portfolio policies, see our Editorial Policies and the Editorial Policy Checklist.

Statistics

For all statistical analyses, confirm that the following items are present in the figure legend, table legend, main text, or Methods section.

n/a	Confirmed	
☐	☒	The exact sample size (*n*) for each experimental group/condition, given as a discrete number and unit of measurement
☐	☒	A statement on whether measurements were taken from distinct samples or whether the same sample was measured repeatedly
☐	☒	The statistical test(s) used AND whether they are one- or two-sided<br>*Only common tests should be described solely by name; describe more complex techniques in the Methods section.*
☐	☒	A description of all covariates tested
☐	☒	A description of any assumptions or corrections, such as tests of normality and adjustment for multiple comparisons
☐	☒	A full description of the statistical parameters including central tendency (e.g. means) or other basic estimates (e.g. regression coefficient) AND variation (e.g. standard deviation) or associated estimates of uncertainty (e.g. confidence intervals)
☐	☒	For null hypothesis testing, the test statistic (e.g. *F*, *t*, *r*) with confidence intervals, effect sizes, degrees of freedom and *P* value noted<br>*Give P values as exact values whenever suitable.*
☒	☐	For Bayesian analysis, information on the choice of priors and Markov chain Monte Carlo settings
☒	☐	For hierarchical and complex designs, identification of the appropriate level for tests and full reporting of outcomes
☐	☒	Estimates of effect sizes (e.g. Cohen's *d*, Pearson's *r*), indicating how they were calculated

Our web collection on statistics for biologists contains articles on many of the points above.

Software and code

Policy information about availability of computer code

Data collection	Data was collected using a proprietary activity based sensor that is in production as a commercial assay. The assay measures the amount of protease activity determined in vitro for serum harvested protease enzymes. The activity was measured using a VarioSkan Lux fluorimeter
Data analysis	Data analysis was performed using Excel for statistical analysis and plotting. Confidence intervals were derived using EpiTools at https://epitools.ausvet.com.au/ciproportion. Algorithm definition was performed using Emerge software proprietary to Liquid Biosciences. The Emerge software is a service product of Liquid Bioscience, Inc. and is not available for release to the public. Liquid Biosciences, Inc. is a commercial entity and their services can be invited by any interested party. Sensitivity and Specificity were calculated using standard formulae.

For manuscripts utilizing custom algorithms or software that are central to the research but not yet described in published literature, software must be made available to editors and reviewers. We strongly encourage code deposition in a community repository (e.g. GitHub). See the Nature Portfolio guidelines for submitting code & software for further information.

Data

Policy information about availability of data

All manuscripts must include a data availability statement. This statement should provide the following information, where applicable:

- Accession codes, unique identifiers, or web links for publicly available datasets
- A description of any restrictions on data availability
- For clinical datasets or third party data, please ensure that the statement adheres to our policy

All source data for all the figures in the main manuscript is available in the Supplementary Data 1.xlsx file available online. Requests for access to the Ensemble Excel model will undergo a prompt review to ensure the request is not subject to any intellectual property or confidentiality obligations. Access to Ensemble Excel model data will be subject to a data transfer agreement. Requests to access this data set should be directed to the corresponding author.

Human research participants

Policy information about studies involving human research participants and Sex and Gender in Research.

Reporting on sex and gender	Both male and female patients were recruited as they presented at the clinic but were not considered in the study design other than reflecting the normal presentation in the risk population. Sex information was collected on an IRB approved Medical History Questionnaire by study staff at each site. The study included 63% male and 37% female participants overall with males contributing 48% samples at KUMC, 52% samples at Marmara University, and 29% samples at Vejle Hospital.
Population characteristics	We analyzed samples collected from individuals at risk for lung cancer. The samples were collected using two different approaches. At two sites, the samples were collected as cohorts of 50 to 80 year old subjects who were current or former smokers (cessation ≤ 15 years) with a smoking history of at least 20 pack-years. Cohort I was defined as subjects with a pathologically confirmed diagnosis of lung cancer that were treatment naïve. All subjects with stage I through stage III NSCLC, or limited stage SCLC diagnosed lung cancer were accepted. Cohort 2 was defined as subjects in the high-risk group for lung cancer based on age and smoking history with no known disease. Subjects were negative for lung cancer as determined by imaging (LDCT) or nodule biopsy that came back negative. A third site was collected in a prospective screening setting. Patients referred on suspicion of lung cancer by primary care physicians were offered enrollment in the study. CT scans are assessed by a specialist in radiology and a specialist in pulmonary medicine to determine whether to continue follow-up CT scans or refer for further diagnostic procedures such as PET-CT, bronchoscopy, or biopsy. Blood was sampled at baseline upon study enrollment. Patients were followed in the study until diagnostic resolution as lung cancer positive or negative.
Recruitment	Written informed consent was obtained from donors and no data allowing identification of patients was provided. Confirmation of diagnosis was performed by research teams at each institution who reviewed medical records to determine the final diagnosis.
Ethics oversight	Research ethics approval was obtained at Kansas University Medical Center and approved by the Human Research Protection Program IRB#: STUDY00144465. The Marmara University study was approved by the Clinical Research Ethics Committee Decision number 898 and the Turkish Ministry of Health; Turkish Medicines and Medical Devices Agency (E-68869993-511.06-570065). The University of Southern Denmark Ethics Committee approved the IRB S-2022014.

Note that full information on the approval of the study protocol must also be provided in the manuscript.

Field-specific reporting

Please select the one below that is the best fit for your research. If you are not sure, read the appropriate sections before making your selection.

☒ Life sciences ☐ Behavioural & social sciences ☐ Ecological, evolutionary & environmental sciences

For a reference copy of the document with all sections, see nature.com/documents/nr-reporting-summary-flat.pdf

Life sciences study design

All studies must disclose on these points even when the disclosure is negative.

Sample size	For our study, we aimed to achieve a 95% confidence interval with the probability of type I error equal to α=0.05. For our study, we determined that a maximum marginal error of 5% was acceptable given the analytic targets of this study (d = 0.05). When the true status or condition is known before or during evaluation, the prevalence may effectively be controlled. In this study we used 25% as the prevalence. The estimates for sensitivity (84%) and specificity (93%) where informed by a development study performed at Hawkeye Bio using a total of 351 commercial samples.Using these values in a power analysis, we estimated the minimal sample size required for this study was 400 participants in order to estimate the specificity. The target prevalence of disease for this study where the true positive population is known, is controlled by sample selection and set at 25%. The study comprised 450 unique patient samples that included 133 serum samples from patients with pathologically confirmed lung cancer and 317 matched controls as described above. A subset of 150 samples selected to reflect the same site and disease distribution were selected for repeated evaluation to determine repeatability. A total of 750 samples were therefore evaluated.

Data exclusions	No data were excluded.
Replication	The precision of the assay was determined and is presented in the paper.
Randomization	We divided the data into training, selection and test sets comprising 1/3 of the samples in each. Care was take to maintain equal representation of disease status, disease stage, trial site, sex, patient age, smoking status, and histology in each set.
Blinding	Allocations were done according to standard procedures in machine learning settings. Model development was performed on the training set, the models were selected in the selection set and validated on the test set.

Reporting for specific materials, systems and methods

We require information from authors about some types of materials, experimental systems and methods used in many studies. Here, indicate whether each material, system or method listed is relevant to your study. If you are not sure if a list item applies to your research, read the appropriate section before selecting a response.

Materials & experimental systems

n/a	Involved in the study	
☒	☐	Antibodies
☒	☐	Eukaryotic cell lines
☒	☐	Palaeontology and archaeology
☒	☐	Animals and other organisms
☐	☒	Clinical data
☒	☐	Dual use research of concern

Methods

n/a	Involved in the study	
☒	☐	ChIP-seq
☒	☐	Flow cytometry
☒	☐	MRI-based neuroimaging

Clinical data

Policy information about clinical studies

All manuscripts should comply with the ICMJE guidelines for publication of clinical research and a completed CONSORT checklist must be included with all submissions.

Clinical trial registration	ClinicalTrials.gov registration was not performed as two of the sites were international and no intervention was performed.
Study protocol	Clinical Trial protocols will be provided upon request. There is a separate protocol for each of the three sites where subjects were recruited.
Data collection	Site HEB.115 was the University of Kansas Medical Center. IRB approval was gained on August 17, 2021 and the study was closed on March 31, 2023. HEB.121 was the University of Marmara School of Medicine, Department of Chest Surgery. The study opened on October 19, 2021 and was concluded on March 29, 2023. Site HEB.130 is Vejle Hospital at The University of Southern Denmark. The study was approved in August 2022 and is ongoing.
Outcomes	Subjects with lung cancer all had pathologically confirmed disease as determined by the clinical teams. The absence of lung cancer was determined by chest CT scan. Any indeterminate results were followed up as decided by the clinical teams administering the patients. Negative lung cancer status was recorded after all necessary diagnostic follow up was completed.
